# Supplementary material for: Code-Free Development and Deployment of Deep Segmentation Models for Digital Pathology
Source: Front Med (Lausanne). 2022 Jan 27;8:816281. doi: 10.3389/fmed.2021.816281 (PMC8829033; doi:10.3389/fmed.2021.816281)
Supplement: Supplementary file 1 [file Data_Sheet_1.pdf]

# *Supplementary Material*

## 1 Supplementary Data

The dataset used in this study consisting of 251 (HE and CD3-stained) colon biopsy WSIs is made openly available at DataverseNO (https://doi.org/10.18710/TLA01U). All source code and a tutorial video can be found in the GitHub repository (https://github.com/andreped/NoCodeSeg).

## 2 Supplementary Figures and Tables

### 2.1 Supplementary Figures

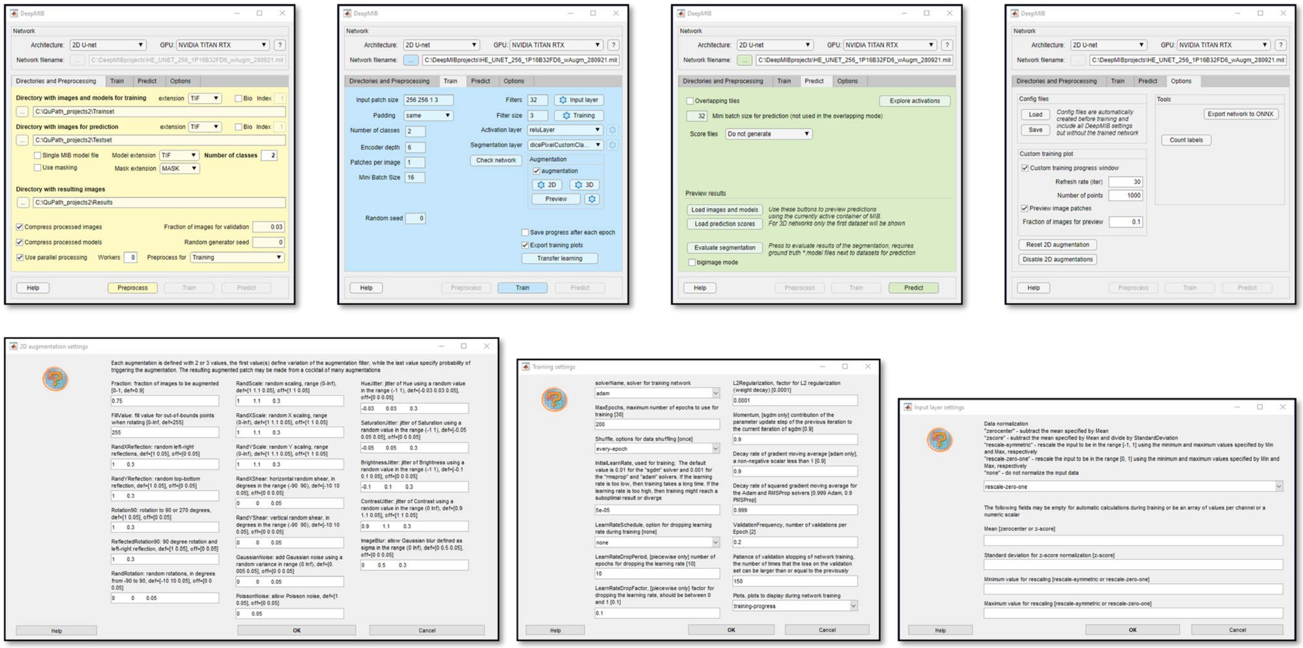

**Supplementary Figure 1.** Example of training hyperparameters used in this study - screenshots from DeepMIB. All trainings had the same hyperparameters indicated by the figure, except the variables indicated in Table 1 in the main paper (i.e., patch size [64x64 – 512x512], number of filters [32 or 64], batch size [16-64]).

### Architecture

| | |
|---|---|
| **Type of available CNN architecture** | - 2D U-Net<br>- 2D SegNet<br>- 3D U-Net<br>- 3D U-Net Anisotropic |

### Padding

| | |
|---|---|
| **Type of the convolution padding** | - same<br>- valid |

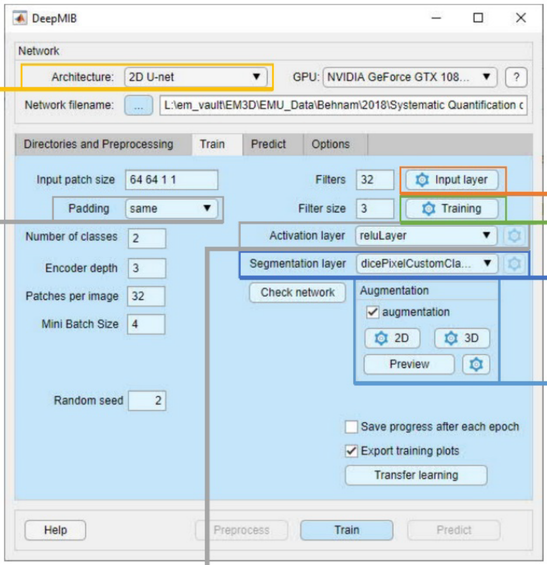


### Input layer settings

| | |
|---|---|
| **Data normalization** | - zerocenter<br>- zscore<br>- rescale-symmetric<br>- rescale-zero-one<br>- none |
| **Mean** | for zeroscenter or zscore |
| **Standard deviation** | for zscore |
| **Minimum value for rescalling** | for rescale-symmetric or rescale-zero-one |
| **Maximum value for rescalling** | for rescale-symmetric or rescale-zero-one |

### Training settings

| | |
|---|---|
| **Network solver** | - adam<br>- rmsprop<br>- sgdm |
| **Max number of Epochs** | *maximum number of epochs to use for training* |
| **Shuffle data** | - every-epoch<br>- once<br>- never |
| **Initial learning rate** | *initial learning rate for training* |
| **Learn rate schedule** | - none<br>- piecewise |
| **Learn rate drop factor** [for piecewise] | *factor for dropping the learning rate during training* |
| **L2 regularization** | *weight decay factor for L2 regularization* |
| **Momentum** [sgdm only] | *contribution of the parameter update step of the previous iteration to the current iteration* |
| **Gradient decay factor** [adam only] | *decay rate of gradient moving average* |
| **Squared gradient decay factor** [adam, rmsprop only] | *decay rate of squared gradient moving average* |
| **Validation frequency** | *number of validations per Epoch* |
| **Validation patience** | *patience of validation stopping of network training* |
| **Plots and Display** | - training-progress<br>- none |

### Activation layer settings

| | |
|---|---|
| **Activation layer** | - relu layer<br>- leaky relu layer<br>- clipped relu layer<br>- elu layer<br>- tanh layer |

### Segmentation layer settings

| | |
|---|---|
| **Segmentation layer** | - pixel classification layer<br>- focal loss layer<br>- dice pixel classification layer<br>- custom dice pixel classification layer |

### Augmentation settings

| operation name | 2D | 3D | operation name | 2D | 3D |
|---|---|---|---|---|---|
| **Fraction of images to augment** | yes | yes | | | |
| **Random X reflection** | yes | yes | **Random X shear** | yes | no |
| **Random Y reflection** | yes | yes | **Random Y shear** | yes | no |
| **Random Z reflection** | NA | yes | **Gaussian noise** | yes | no |
| **Rotation 90 degrees** | yes | yes | **Poisson noise** | yes | no |
| **Reflected rotation 90 degrees** | yes | yes | **Hue jitter** | yes | no |
| **Random rotation** | yes | no | **Saturation jitter** | yes | no |
| **Random scaling** | yes | no | **Brightness jitter** | yes | no |
| **Random X scaling** | yes | no | **Contrast jitter** | yes | no |
| **Random Y scaling** | yes | no | **Image blur** | yes | no |

**Supplementary Figure 2:** The graphical user interface of DeepMIB with explanation of the available options for training of 2D U-Net, 2D SegNet, and 3D U-Net deep segmentation networks.

## 2.2 Supplementary Tables

**Supplementary Table 2. Estimated mean IoU and difference compared to the U-Net 512x512 algorithm on the HE and CD3 datasets using mixed linear regression.**

| | | Estimated margin mean IOU | | | Comparison to HE/CD3 U-Net 512x512 | | | |
|---|---|---|---|---|---|---|---|---|
| Dataset | Architecture and hyperparameters | mean | 95 % CI (lower limit) | 95 % CI (upper limit) | Estimated difference in IOU | 95 % CI (lower limit) | 95 % CI (upper limit) | p-value |
| HE | **U-Net 512x512, 32 filters, 16 batch** | **0.952** | **0.944** | **0.960** | **0.000** | **.** | **.** | **.** |
| | U-Net 256x256, 32 filters, 16 batch | 0.936 | 0.919 | 0.953 | -0.016 | -0.028 | -0.003 | 0.013 |
| | U-Net 256x256, 32 filters, 32 batch | 0.932 | 0.916 | 0.948 | -0.019 | -0.030 | -0.009 | $2.16 \times 10^{-4}$ |
| | U-Net 256x256, 64 filters, 32 batch | 0.932 | 0.916 | 0.949 | -0.019 | -0.030 | -0.008 | $5.07 \times 10^{-4}$ |
| | U-Net 128x128, 32 filters, 16 batch | 0.929 | 0.911 | 0.947 | -0.023 | -0.036 | -0.010 | $6.82 \times 10^{-4}$ |
| | U-Net 64x64, 32 filters, 16 batch | 0.921 | 0.902 | 0.940 | -0.031 | -0.044 | -0.017 | $6.27 \times 10^{-6}$ |
| | SegNet 512x512, 32 filters, 16 batch | 0.925 | 0.912 | 0.938 | -0.027 | -0.034 | -0.019 | $4.00 \times 10^{-12}$ |
| | SegNet 256x256, 32 filters, 16 batch | 0.935 | 0.921 | 0.949 | -0.017 | -0.025 | -0.009 | $5.88 \times 10^{-5}$ |
| | SegNet 128x128, 32 filters, 16 batch | 0.902 | 0.882 | 0.922 | -0.050 | -0.064 | -0.035 | $6.03 \times 10^{-11}$ |
| CD3 | **U-Net 512x512, 32 filters, 16 batch** | **0.958** | **0.948** | **0.968** | **0.000** | **.** | **.** | **.** |
| | U-Net 256x256, 32 filters, 16 batch | 0.933 | 0.913 | 0.953 | -0.025 | -0.038 | -0.011 | $2.40 \times 10^{-4}$ |
| | SegNet 512x512, 32 filters, 16 batch | 0.919 | 0.905 | 0.934 | -0.038 | -0.048 | -0.028 | $6.81 \times 10^{-14}$ |
| | SegNet 256x256, 32 filters, 16 batch | 0.899 | 0.883 | 0.915 | -0.058 | -0.068 | -0.049 | $2.39 \times 10^{-32}$ |

*Each architecture was compared to the single best performing architecture (U-Net 512x512 32 filters, depth 6, 16 batch, indicated in* ***bold****) for the HE/CD3 datasets separately, and the estimated mean difference is presented together with 95 % CI and p-values using two-level mixed regression models, where architecture is level 1, image patch is level 2, and with robust variance estimates clustered by WSI and a random intercept for patch.*
